# Effects of Enzyme–Microbe Co-Fermented *Ganoderma lucidum* Spent Substrate on Growth Performance, Apparent Nutrient Digestibility, Organ Indices, and Gut Microbiota in Yellow-Feathered Broilers

**DOI:** 10.3390/ani16060949

**Published:** 2026-03-18

**Authors:** Bo Fan, Mengyun Li, Zhifang Shi, Xuanyang Li, Tongshuai Liu, Pu Cheng, Lei Xi

**Affiliations:** 1 Animal Science, Xinjiang Agricultural University, No. 311 Nongda East Road, Urmuqi 830052, China; fbbb419@163.com; 2 Animal Science, Henan University of Animal Husbandry and Economy, No. 6 Longzihu North Road, Zhengzhou 450046, China; limengyun1@163.com (M.L.); shizhifang83158@163.com (Z.S.); 201006@hnuahe.edu.cn (X.L.); 80971@hnuahe.edu.cn (T.L.); chengpu1@163.com (P.C.)

**Keywords:** yellow-feathered broilers, enzyme–microbe co-fermented *Ganoderma lucidum* spent substrate, growth performance, apparent nutrient digestibility, organ indices, cecal microbiota, dietary supplement

## Abstract

The effects of dietary supplementation of enzyme–microbe co-fermented Ganoderma lucidum spent substrate (EFGLS) on growth performance, apparent nutrient digestibility, organ indices, and cecal microbiota in yellow-feathered broilers are investigated. Healthy broilers (450 individuals of 22 days age) of similar body weight were randomly assigned to three dietary treatments (five replicates/treatment, 30 birds/replicate). A control group received a corn–soybean meal-based basal diet; treatments received diets containing 1.5% or 3.0% EFGLS. Over six weeks, treatment-group broilers exhibited significantly greater average daily gain and a lower feed-to-gain ratio compared with the control group (*p* < 0.001); differences in apparent nutrient digestibility in EFGLS-supplemented groups were not significant. A thymus index was significantly higher in the 1.5% than 3.0% EFGLS group (*p* < 0.05); Pielou’s evenness, Shannon, and Simpson indices of cecal microbiota were significantly higher in the 3.0% EFGLS group than control group (*p* < 0.05); and a dominance index was significantly higher in the control group than in treatment groups. Under study conditions, dietary supplementation with EFGLS improved growth performance in broilers, associated with favorable changes in apparent nutrient digestibility, immune organ development, and cecal microbial community structure. Accordingly, we recommend a dietary supplementation level of 1.5% EFGLS.

## Simple Summary

This study evaluated the use of enzyme–microbe co-fermented Ganoderma lucidum spent substrate (EFGLS) as a feed additive for yellow-feathered broiler chickens. In a six-week feeding trial, broilers were fed diets supplemented with 1.5% or 3.0% EFGLS, partially replacing corn and soybean meal in a conventional basal diet. Both supplementation levels improved growth performance and feed efficiency compared with a control diet, while apparent nutrient utilization also trended upwards. A dietary inclusion level of 1.5% EFGLS was associated with improved thymus development, whereas the 3.0% supplementation level increased cecal microbial diversity. Notably, beneficial dominant microbial populations were more consistently enriched in broilers fed the 1.5% EFGLS supplemented diet. Overall, EFGLS represents a promising functional feed ingredient for broiler production, and under the conditions of this study, a supplementation level of 1.5% EFGLS is recommended.

## 1. Introduction

With increased demand for high-quality animal protein, poultry meat has gained widespread consumer acceptance because of its low fat content, high protein value, and relatively affordable price. The rapid expansion of the poultry industry has consequently intensified challenges related to nutrient utilization efficiency and environmental sustainability.

*Ganoderma lucidum* is a traditional Chinese precious medicinal material that is rich in bioactive compounds, including polysaccharides, triterpenoids, phenolic substances, and trace elements, etc., that have substantial pharmacological value [1]. According to the China Edible Fungi Association, the total production of *G. lucidum* in China reached approximately 150,000 tons in 2023 [2]. Alongside large-scale production, substantial quantities of *G. lucidum* spent substrate are generated as by-products. This spent substrate, derived from spent mushroom substrates, contains abundant organic matter and mineral elements [3], and has been recognized as a promising feed resource for livestock and poultry [4]. Poultry are characterized by a relatively short digestive tract and the absence of separation between feces and urine, which often results in incomplete digestion of dietary nutrients. As a result, poultry manure contains high levels of organic matter, and the subsequent microbial fermentation of this excreta produces malodorous gases such as ammonia and hydrogen sulfide that create significant environmental problems [5]. Previous studies have shown that dietary inclusion of 3% *G. lucidum* spent substrate to laying hens reduced ammonia and hydrogen sulfide emissions from manure [6], while supplementation in laying ducks decreased serum lipid levels [7]. In ruminants, the inclusion of fermented *G. lucidum* mycelia enhances immune function and antioxidant capacity in dairy cows [8].

Microbial fermentation is an effective strategy for the valorization of agricultural by-products and for alleviating pressure on conventional feed resources. Fermentation can degrade fibrous components, reduce anti-nutritional factors, and enrich substrates with bioactive metabolites such as peptides, enzymes, and beneficial microorganisms. These fermentation-derived compounds may influence gut microbial ecology and host nutrient metabolism [9]. Numerous studies have demonstrated that fermented feeds can improve growth performance, modulate intestinal microbiota, reduce nitrogen and phosphorus excretion, and mitigate environmental pollution in poultry production systems [10,11]. The application of fermentation technology to *G. lucidum* spent substrate can potentially enhance its nutritional value and functional properties. Positive effects of fermented *Flammulina velutipes* spent substrate on growth and health parameters have already been reported in meat sheep [12]. Anti-nutrient substances are compounds originating in most animal feed materials that are toxic to animals and limit the accessibility of nutrients to the animal body [13]. Combined microbial–enzyme fermentation can efficiently degrade anti-nutritional factors. Based on solid-state fermentation technology, this approach is generally classified into one- and two-step fermentation processes, and it is considered to be a key technique in the development and utilization of unconventional feed resources [14]. Although the underlying mechanisms remain incompletely understood, this approach has attracted considerable research attention because of its favorable fermentation performance.

While fermentation of other agricultural spent substrate has been investigated [15,16], data on fermented *G. lucidum* spent substrate in broilers, especially yellow-feathered broilers, is limited. In particular, for these broilers, its effects on growth performance, nutrient utilization, immune organ development, and cecal microbiota are not fully known. Therefore, we investigate the effects of supplementing diet with two levels of enzyme–microbe co-fermented *G. lucidum* spent substrate (EFGLS) on growth performance, nutrient utilization, immune organ development, and cecal microbiota in yellow-feathered broilers.

## 2. Materials and Methods

### 2.1. Preparation of Fermented Ganoderma lucidum Spent Substrate

EFGLS was produced by Henan dong-fangjianbiotech Co., Ltd. (Puyang, China). The fermentation procedure and microbial strain selection were performed in accordance with Chen and Heng [17,18], with minor modifications. The compound probiotic preparation was provided by the laboratory of Henan dongfangjianbiotech Co., Ltd., and the compound enzyme preparation (Item number: Xinyangmei 1819E11) was purchased from Smistyle (Neiqiu) Bio-tech Co., Ltd. (Xingtai, China). The collected *G. lucidum* spent substrate (the spent cultivation substrate remaining after *G. lucidum* fruiting body harvest, consisting primarily of sawdust, wheat bran, sugarcane bagasse, residual mycelia, and their metabolic products) was oven-dried at 60–70 °C to constant weight then ground to pass through a 40-mesh sieve. The resulting powder was mixed with corn flour, soybean meal, and wheat bran at a ratio of 7:1:1:1 (*w*/*w*). After thorough mixing, its moisture content was adjusted to approximately 50%. Subsequently, 2% (*w*/*w*) of a compound probiotic preparation and 0.02% (*w*/*w*) of a compound enzyme preparation were added, and the mixture was homogenized, packed into breathable fermentation bags, de-aerated, and incubated at 37 °C for 7 days to complete fermentation.

The compound probiotic preparation mainly consisted of *Bacillus subtilis* (viable count ≥ 2.5 × 10^8^ CFU/g), *Saccharomyces cerevisiae* (≥6.0 × 10^8^ CFU/g), and *Lactobacillus plantarum* (≥2.0 × 10^9^ CFU/g). The compound enzyme preparation contained xylanase (≥20,000 U/g), β-mannanase (≥1200 U/g), β-glucanase (≥700 U/g), amylase (≥200 U/g), protease (≥1000 U/g), and cellulase (≥100 U/g). A synergistic interaction exists between microbial inoculants and enzyme preparations [19]. The compound enzyme breaks down the starch, non-starch polysaccharides, cellulose, and structural proteins in the fungus spent substrate, providing energy for the fungus, accelerating the start of fermentation, and providing microbial nitrogen sources. Ultimately, this improves the fermentation quality, making the nutrients easier for animals to absorb and utilize.

### 2.2. Birds, Diets and Experiment

A total of 450 mixed-sex 22-day-old yellow-feathered broilers of similar body weight were randomly assigned to three dietary treatments. Each treatment included five replicates, with 30 birds per replicate, and each replicate served as an independent experimental unit and was housed individually. Dietary groups consisted of (1) a corn–soybean meal basal diet (Control); (2) basal diet supplemented with 1.5% EFGLS partially replacing corn and soybean meal (1.5% EFGLS); and (3) basal diet supplemented with 3% EFGLS partially replacing corn and soybean meal (3% EFGLS). Inclusion levels of 1.5% and 3.0% EFGLS were selected to represent moderate and relatively high supplementation levels in the diet. Previous studies investigating the application of fermented spent substrates and related by-products in broiler nutrition have mostly investigated inclusion levels of 0.5–3.0% [20,21], with higher supplementation levels reported to negatively affect broiler growth performance [21]. Based on these findings and discussions within our research group, we considered inclusion levels of 1.5% and 3.0% to be appropriate to evaluate potential dose-dependent effects while maintaining practical feed formulation. All experimental diets were formulated to be isonitrogenous and isoenergetic. The basal diet was formulated according to the Feeding Standard of Chicken (NY/T 33–2004); ingredient composition and nutrient levels are detailed in Table 1. The experimental period lasted for six weeks, from 22–63 days of age.

### 2.3. Animal Management

The feeding trial was performed at an experimental poultry facility. Throughout the experiment, broilers had ad libitum access to feed and water. The ambient temperature was maintained at 27–30 °C during the first week, then reduced by 2–3 °C per week until reaching approximately 20 °C, where it was maintained until the end of the experiment. Natural ventilation was provided, and birds were vaccinated according to standard farm immunization procedures. Manure was removed regularly, and feed intake and mortality were recorded daily.

### 2.4. Measurements and Sample Collection

#### 2.4.1. Growth Performance

Broilers were weighed on days 22, 42, and 62 of age after feed withdrawal, on a replicate basis. Average daily gain (ADG) was calculated accordingly. Feed intake was recorded on a replicate basis throughout the experimental period to calculate average daily feed intake (ADFI). The feed-to-gain ratio (F/G) was calculated using ADFI and ADG.

#### 2.4.2. Apparent Nutrient Digestibility

From days 60–62, excreta samples were collected continuously for three consecutive days on a replicate basis. Samples were thoroughly mixed, acidified with 10% dilute hydrochloric acid to fix nitrogen, air-dried, ground, and then passed through a 40-mesh sieve before analysis. Crude protein contents in feed and excreta were determined according to GB/T 6432–2018 [22], crude fat following GB/T 6433–2006 [23], sulfur following GB/T 17776-2016 [24], crude ash following GB/T 6438–2007 [25], and total phosphorus following GB/T 6437–2018 [26]. Acid-insoluble ash (AIA) was determined following GB/T 23742–2009 [27] and used as an internal marker to calculate apparent nutrient digestibility. Apparent nutrient digestibility was calculated using Formula (1):

Apparent nutrient digestibility (%) = 100% − (AIA content in feed × nutrient content in excreta)/(nutrient content in feed × AIA content in excreta) × 100%(1)

#### 2.4.3. Organ Indices

On day 63, one broiler of body weight close to the replicate mean was selected from each replicate after a 12 h feed withdrawal (with free access to water) and humanely slaughtered. The heart, liver, thymus, spleen, bursa of Fabricius, gizzard, proventriculus, and intestines were excised, and adherent fat was removed before weighing. Organ indices were calculated as the ratio of organ weight to body weight. Organ indices were calculated using Formula (2):

Organ index (%) = Organ weight (g)/Live body weight (g) × 100%(2)

#### 2.4.4. Cecal Microbiota Analysis

After slaughter, cecal contents were aseptically collected into sterile cryogenic tubes, immediately frozen in liquid nitrogen, and stored at −80 °C until analysis. High-throughput sequencing of the 16S rRNA gene was performed by Novogene Co., Ltd. (Beijing, China). Amplification of the 16S rRNA gene targeted the V3–V4 regions and used primers 341F (5′-CCTAYGGGRBGCASCAG-3′) and 806R (5′-GGACTACNNGGGTATCTAAT-3′). Bioinformatic analyses were performed using NovoMagic (Novogene Co., Ltd., Beijing, China).

### 2.5. Statistical Analysis

All data were analyzed using SPSS software (version 27.0; SPSS Inc., Chicago, IL, USA). One-way analysis of variance (ANOVA) was performed to evaluate differences among treatments, and Duncan’s multiple range tests were used for post hoc comparisons. Differences were considered highly significant at *p* < 0.01 and significant at *p* ≤ 0.05.

## 3. Results

### 3.1. Effects of EFGLS on Growth Performance

From 43–63 days of age, the final body weights and ADGs of broilers in 1.5% and 3% EFGLS groups were higher than those of the control (*p* < 0.001), while F/G was markedly reduced (*p* = 0.001) (Table 2). Over the entire experimental period (22–63 days of age), broilers in 1.5% and 3% EFGLS treatments exhibited greater final body weights and ADGs than controls (*p* < 0.001), and a lower F/G (*p* < 0.05).

### 3.2. Effects of EFGLS on Apparent Nutrient Digestibility

No significant differences in apparent nutrient digestibility were observed among groups (*p* > 0.05) (Table 3). However, compared with control broilers, those fed diets supplemented with EFGLS had numerically higher apparent digestibility of all measured nutrients.

### 3.3. Effects of EFGLS on Organ Indices

The thymus index in the 1.5% EFGLS group was higher than that in the 3% EFGLS group (*p* < 0.05) (Table 4). For indices of other organs there were no significant differences among groups.

### 3.4. Effects of EFGLS on Cecal Microbiota

#### 3.4.1. Alpha Diversity

Pielou’s evenness and Shannon indices of cecal microbiota in the 1.5% EFGLS treatment were higher than those in the control (*p* < 0.05) (Table 5). Simpson’s index was lower, and the dominance index was higher in the control than in supplementation groups (*p* < 0.05). No significant differences were observed among treatments in Chao1 or observed features indices.

#### 3.4.2. Amplicon Sequence Variant (ASV) Clustering Analysis

**Figure 1:**
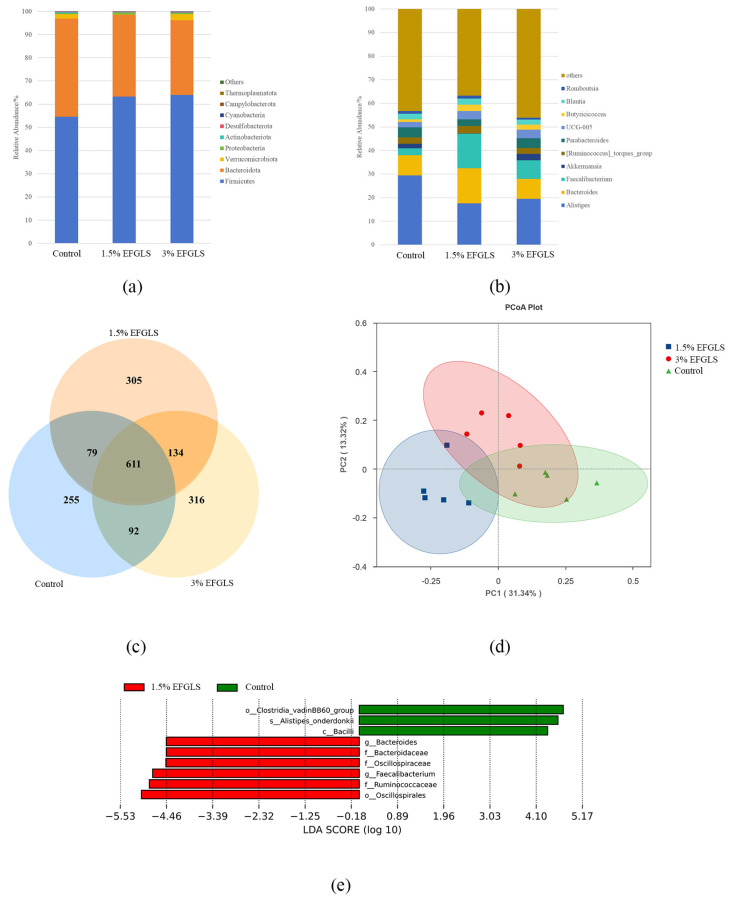
Effects of dietary EFGLS supplementation on cecal microbial community structure and composition in 63-day-old broilers (control; 1.5% EFGLS; and 3% EFGLS groups). Relative abundance of the top 10 bacterial (**a**) phyla and (**b**) genera; (**c**) Venn diagram illustrating shared and unique ASVs among dietary treatment groups; (**d**) principal coordinates analysis based on Bray–Curtis distances revealing differences among treatments; (**e**) linear discriminant analysis effect size identifying differentially enriched bacterial taxa among dietary treatments (LDA score > 4).

A total of 611 ASVs were shared among the three groups (Figure 1c). The control group had 1037 ASVs, of which 255 were unique (24.59%). Group 1.5% EFGLS had 1129 ASVs, of which 305 were unique (27.02%), and the 3% EFGLS group had 1153 ASVs, of which 316 were unique (27.41%).

#### 3.4.3. Taxonomic Composition at Phylum and Genus Levels

At the level of phylum (Figure 1a), *Firmicutes* and *Bacteroidota* were dominant in the cecal microbiota of all groups (Table 6). The relative abundance of *Bacteroidota* in the control group was higher than that in the 3% EFGLS group (*p* < 0.05), while that of *Actinobacteriota* was higher in the control group than in either supplementation group (*p* < 0.05). The five most abundant genera across all groups were *Alistipes*, *Bacteroides*, *Faecalibacterium*, *Akkermansia*, and *[Ruminococcus]*_torques_group (Figure 1b). The relative abundance of *Alistipes* was higher in the control group than in either supplementation group (*p* < 0.05). The 1.5% EFGLS group had higher relative abundances of *Faecalibacterium* compared with the control and 3% EFGLS groups (*p* < 0.05).

#### 3.4.4. Beta Diversity Analysis

Principal coordinates 1 (PC1) and PC2 explained 31.34% and 13.32% of the total variation, respectively (Figure 1d). Distinct clusters were observed between control and supplementation groups. Adonis analysis revealed community variation to be 38% between the 1.5% EFGLS and control groups (R^2^ = 0.38, *p* < 0.01), 25% between the 3% EFGLS and control groups (R^2^ = 0.25, *p* < 0.01), and 28% between the 1.5% EFGLS and 3% EFGLS groups (R^2^ = 0.28, *p* > 0.01).

#### 3.4.5. Differential Taxa Analysis

Linear discriminant analysis effect size (LEfSe) analysis with an LDA score threshold of 4 identified distinct microbial biomarkers among treatments (Figure 1e). Differentially abundant taxa were identified in the control and 1.5% EFGLS groups.

## 4. Discussion

Fermented feed ingredients have been widely reported to enhance animal growth performance. Previous studies have demonstrated that dietary supplementation with fermented mushroom spent substrate improves ADG in pigs [28], sheep [29], and broilers [30], while liquid fermentation products of *G. lucidum* have also shown growth-promoting effects in broilers [31]. Consistent with these findings, we report the inclusion of EFGLS in the diet of yellow-feathered broilers benefits their growth performance.

The growth-promoting effects of EFGLS were more evident during later growth stages, consistent with Xu et al. [32], who reported fermented feeds to exert stronger effects during latter growth phases of animals. Although ADFI was not significantly affected, the numerically higher feed intake observed in supplementation groups may be attributed to improved diet palatability resulting from fermentation.

The digestibility and utilization of dietary nutrients in poultry are influenced both by the birds’ digestive capacity and the nutritional composition and processing characteristics of the diet. Appropriate fermentation techniques improve feed quality [33], enhance the utilization efficiency of certain feed ingredients [34], reduce the content of anti-nutritional factors in raw materials [35], improve protein and lipid utilization, and promote mineral and amino acid absorption by modulating gut pH and microbial balance [36,37]. However, we found that supplementation with EFGLS did not significantly affect apparent nutrient digestibility. Although this finding differs from most previous reports, supplementation with fermented feed does not always significantly affect nitrogen digestibility in chickens [38]. This may be due to the relatively modest influence of EFGLS on apparent nutrient digestibility, with any beneficial effect primarily reflected in improvements in intestinal health. Moreover, variability in the recovery of AIA and the analytical procedures involved may affect the sensitivity of apparent digestibility measurements. Considered alongside growth-performance results, dietary supplementation with EFGLS improved the productive performance of broilers without exerting adverse effects on nutrient digestibility. Moreover, the partial replacement of conventional feed ingredients may help to reduce feeding costs, indicating its potential applicability in practical poultry production.

Organ indices are commonly used as indicators of immune development and digestive function in animals. Among them, the bursa of Fabricius, thymus, and spleen are the principal immune organs. Because viral infection in chickens can induce atrophy of immune organs [39], the state of their development can, to an extent, reflect immune competence in poultry. The thymus is important in the differentiation and maturation of T lymphocytes, and its development is highly sensitive to nutritional status and gut-derived immunoregulatory signals. Fermented feed ingredients can positively modulate immune organ development in poultry [40]. We report the thymus index in the 1.5% EFGLS group to trend upwards against the control. Although this difference was not statistically significant, it may indicate that a moderate level of fermented substrate supplementation benefits thymic development and immune maturation. In contrast, a significantly lower thymus index occurred in broilers that received the 3% supplementation level, suggesting that excessive inclusion of EFGLS may be of no further benefit, and may even compromise immune organ development. Similar dose-dependent effects have been reported for other fermented feed ingredients [41]. *Ganoderma lucidum* is rich in bioactive components such as triterpenoids and saponins—compounds with immunomodulatory activities [4]. However, excessive accumulation of these bioactive substances in vivo may exert cytotoxic effects on various cell lines, significantly reducing cell viability [42]. Excessive dietary supplementation with EFGLS may lead to the accumulation of these bioactive compounds in broilers and exert inhibitory effects on the development of immune organs, possibly explaining the reduced thymus index observed in the 3% EFGLS treatment. The underlying mechanisms, however, remain unclear and warrant further investigation.

The heart plays an important role in meeting the increased metabolic demands associated with rapid growth, while the liver serves as the primary organ for nutrient metabolism and detoxification. The small intestine is the primary site of digestion and nutrient absorption in chickens. Fermented feed can improve digestive capacity by enhancing intestinal structure in broilers [43]. However, we report that supplementation with EFGLS did not significantly affect intestine, heart, and liver indices.

Alpha diversity indices are widely used to evaluate microbial richness and evenness, which are closely linked to ecosystem stability and functional redundancy of the intestinal microbiota [44]. We report dietary supplementation with EFGLS to be generally associated with increased microbial richness and a more even cecal microbial community, suggesting a beneficial modulation of gut microbial ecology. Similar improvements in intestinal microbial diversity have been reported in poultry fed fermented diets at appropriate inclusion levels [45]. The observed changes in alpha diversity were further supported by ASV analysis, indicating that EFGLS supplementation reshaped cecal microbial composition. The greater overlap in ASVs between the two EFGLS-supplemented groups compared with the control suggests that EFGLS may exert a directional effect on microbial community assembly and selectively enrich specific microbial members. The specific functional roles of these taxa require further investigation.

Principal coordinates analysis revealed clear separations between the control and EFGLS-supplemented groups. These differences were further supported by Adonis analysis, confirming that EFGLS supplementation altered overall cecal microbial community structure. Such alterations may be attributed to the combined effects of fermentation-derived enzymes, probiotics, and bioactive compounds present in *G. lucidum* spent substrate (we did not investigate the underlying mechanisms).

Previous studies have shown that dietary inclusion of mushroom spent substrates or herbal by-products can modify dominant microbial phyla and genera in ruminants and monogastric animals [28,46]. At the taxonomic level, *Firmicutes* and *Bacteroidota* dominated the cecal microbiota across all treatments, consistent with previous reports [47]. Although the interpretation of the F/B ratio remains controversial, associations between higher F/B ratios and improved growth performance have been reported for poultry [48,49]. We observed a similar pattern, suggesting that EFGLS supplementation may influence the relative abundance of *Firmicutes* and *Bacteroidota*, potentially reflecting changes in energy utilization and metabolic efficiency. Combined with species abundances at phylum and genus levels and LEfSe analysis, EFGLS supplementation, particularly at 1.5%, favored enrichment of fiber-degrading and short-chain fatty acid-producing taxa, including *Bacteroides*, *Faecalibacterium*, Oscillospiraceae, and Ruminococcaceae. These microbial groups play key roles in polysaccharide degradation, butyrate production, and maintenance of intestinal barrier integrity [50,51,52]. Short-chain fatty acids produced by microbial fermentation play important roles in regulating immune homeostasis and development of immune organs [53,54]. This may partially explain the association between changes in dominant cecal microbiota and the improved thymus development observed in the low-dose EFGLS supplementation group. However, some members of *Bacteroides* are also involved in tryptophan metabolism, producing indole and skatole [55], which could lead to deterioration of the housing environment and negatively affect broiler welfare and performance. Therefore, further studies are required to identify functionally distinct *Bacteroides* species or strains in the broiler cecum and to clarify their specific roles. We report such enrichment in the 1.5% EFGLS group but not the 3% EFGLS group, indicating a potential dose-dependent response. Similar findings have been reported in broilers and other monogastric animals, where excessive inclusion of fermented feeds attenuated the proliferation of beneficial taxa or reduced microbial diversity [40,56,57].

## 5. Conclusions

Dietary supplementation with EFGLS improved growth performance, enhanced thymus development, and modulated cecal microbial composition in yellow-feathered broilers. Based on overall results, a dietary inclusion level of 1.5% EFGLS is recommended under the conditions of this study. As a fermented agricultural by-product, EFGLS offers advantages in terms of resource reutilization and sustainability, demonstrating practical potential as an alternative feed ingredient in commercial broiler production.

## Figures and Tables

**Table 1:** Ingredient and calculated nutrient value of basal diets.

Ingredients (%)	Treatments ^1^		
	Control	1.5% EFGLS	3% EFGLS
Corn	59	5.85	58
Soybean meal	34	33	32
EFGLS	0	1.5	3
Soybean oil	3	3	3
Premix ^2^	4	4	4
Total	100	100	100
Calculated nutrient value			
Crude protein (%)	20.04	19.77	19.49
Calcium (%)	1.01	1.01	1.01
Phosphorus (%)	0.36	0.36	0.35
Lysine (%)	1.20	1.18	1.15
Methionine (%)	0.37	0.36	0.36
Metabolizable energy (MJ/kg)	12.47	12.50	12.53

^1^ Treatments: control diet (Control), and control diet supplemented with 1.5% and 3% EFGLS. ^2^ The premix provided the following IU/kg of diets for vitamins A (10,000), D_3_ (3500), and E (28); and mg/kg of diet for vitamins K_3_ (2.2), B_1_ (2.5), B_2_ (3.5), B_6_ (3.0), and B_12_ (0.025), and for biotin (0.25), folic acid (0.8), nicotinic acid (32.0), D-calcium pantothenate (12.0), Fe (90), Cu (18), Mn (90), Zn (70), Se (0.3), and I (0.5).

**Table 2:** Growth performance of broilers fed different dietary EFGLS supplementation levels.

Items	Treatments ^1^			SEM ^2^	*p*-Value
	Control	1.5% EFGLS	3% EFGLS		
22–42 d					
Initial BW (g)	255.047	257.620	254.247	1.504	0.666
Final BW (g)	816.400	851.767	823.300	9.066	0.255
ADG (g)	28.070	29.706	28.452	0.382	0.194
ADFI (g)	66.364	68.384	67.950	0.631	0.418
F/G	2.366	2.302	2.390	0.022	0.244
43–63 d					
Initial BW (g)	845.680	882.360	856.800	9.255	0.269
Final BW (g)	1662.496 ^b^	1817.680 ^b^	1781.296 ^b^	19.751	<0.001
ADG (g)	40.840 ^b^	46.766 ^a^	46.224 ^a^	0.827	<0.001
ADFI (g)	116.792	123.178	118.622	1.380	0.151
F/G	2.858 ^a^	2.638 ^b^	2.568 ^b^	0.040	0.001
22–63 d					
Initial BW (g)	255.047	257.620	254.247	1.504	0.666
Final BW (g)	1662.496 ^b^	1817.680 ^a^	1781.296 ^a^	19.751	<0.001
ADG (g)	35.188 ^b^	39.002 ^a^	38.174 ^a^	0.489	<0.001
ADFI (g)	91.578	95.780	93.288	0.878	0.144
F/G	2.604 ^a^	2.458 ^b^	2.444 ^b^	0.030	0.035

^ab^ Means in the same row with different superscripts differ significantly at *p* ≤ 0.05. ^1^ Treatments: control diet (Control), and diets supplemented with EFGLS at 1.5% and 3%. ^2^ SEM, standard error of the mean (*n* = 15).

**Table 3:** Apparent nutrient digestibility of broilers at different dietary EFGLS supplementation levels.

Items (%)	Treatments ^1^			SEM ^2^	*p*-Value
	Control	1.5% EFGLS	3% EFGLS		
Crude protein	54.509	55.218	55.081	0.297	0.621
Crude fat	81.685	83.007	83.487	0.405	0.174
Phosphorus	51.162	53.871	51.395	0.988	0.497
Sulfur	52.196	55.314	53.989	0.780	0.279

Acid-insoluble ash (AIA) was used as an internal marker to calculate apparent nutrient digestibility. ^1^ Treatments: control diet (Control), and diets supplemented with 1.5% and 3% EFGLS. ^2^ SEM, standard error of the mean (*n* = 15).

**Table 4:** Organ indices of broilers at different dietary EFGLS supplementation levels.

Items (%)	Treatments ^1^			SEM ^2^	*p*-Value
	Control	1.5% EFGLS	3% EFGLS		
Heart	0.495	0.644	0.549	0.029	0.092
Liver	1.593	1.712	1.585	0.033	0.216
Thymus	0.534 ^ab^	0.637 ^a^	0.359 ^b^	0.049	0.049
Spleen	0.123	0.151	0.151	0.007	0.176
Bursa of Fabricius	0.245	0.267	0.295	0.024	0.739
Gizzard	1.476	1.370	1.814	0.104	0.202
Glandular stomach	0.369	0.353	0.402	0.017	0.537
Intestine	3.370	3.816	3.731	0.095	0.125

^ab^ Means within a row with different superscript letters differ significantly at *p* ≤ 0.05. ^1^ Treatments: control diet (Control), and diets supplemented with 1.5% or 3% EFGLS. ^2^ SEM, standard error of the mean (*n* = 15).

**Table 5:** Alpha diversity indices of cecal microbiota in broilers at different dietary EFGLS supplementation levels.

Items	Treatments ^1^			SEM ^2^	*p*-Value
	Control	1.5% EFGLS	3% EFGLS		
Chao1	513.195	571.786	610.664	17.364	0.057
Dominance	0.058 ^a^	0.037 ^b^	0.028 ^b^	0.005	0.013
Observed features	510.400	567.200	607.600	17.133	0.053
Pielou’s	0.668 ^b^	0.698 ^ab^	0.736 ^a^	0.011	0.022
Shannon	6.009 ^b^	6.385 ^ab^	6.804 ^a^	0.125	0.019
Simpson	0.942 ^b^	0.963 ^a^	0.972 ^a^	0.005	0.013

^ab^ Means within a row with different superscripts differ significantly at *p* ≤ 0.05. ^1^ Treatments: control diet (Control), and diets supplemented with 1.5% or 3% EFGLS. ^2^ SEM, standard error of the mean (*n* = 15).

**Table 6:** Relative abundance of the top five cecal bacterial taxa (phyla and genera) in broilers fed different EFGLS supplementation levels.

Taxon (%)	Treatments ^1^			SEM ^2^	*p*-Value
	Control	1.5% EFGLS	3% EFGLS		
*Firmicutes*	54.650	63.307	64.045	1.868	0.061
*Bacteroidota*	42.306 ^a^	35.338 ^ab^	32.157 ^b^	1.796	0.048
*Verrucomicrobiota*	1.859	0.067	2.688	0.641	0.246
*Proteobacteria*	0.344	0.852	0.477	0.128	0.258
*Actinobacteriota*	0.460 ^a^	0.097 ^b^	0.154 ^b^	0.065	0.034
*Alistipes*	29.474 ^a^	17.617 ^b^	19.444 ^b^	2.094	0.030
*Bacteroides*	8.576	14.908	8.534	1.289	0.054
*Faecalibacterium*	2.902 ^b^	14.646 ^a^	7.866 ^b^	1.597	0.002
*Akkermansia*	1.859	0.067	2.688	0.641	0.246
*[Ruminococeus]*_torques_group	2.726	3.220	2.502	0.465	0.835

^ab^ Means within a row with different superscripts differ significantly at *p* ≤ 0.05. ^1^ Treatments: control diet (Control), and diets supplemented with 1.5% or 3% EFGLS. ^2^ SEM, standard error of the mean (*n* = 15).

## Data Availability

The original data presented in the study are openly available in Mendeley Data at DOI: 10.17632/jrm62mnkhy.1.
